# How should we measure population-level inbreeding depression? Impacts of standing genetic associations between selfing rate and deleterious mutations

**DOI:** 10.3389/fpls.2024.1379730

**Published:** 2024-07-09

**Authors:** Kuangyi Xu

**Affiliations:** Department of Ecology and Evolutionary Biology, The University of Toronto, Toronto, ON, Canada

**Keywords:** selfing, mating system, inbreeding depression, genetic association, genetic variation, quantitative genetics

## Abstract

Inbreeding depression (ID) is a major selective force during mating system evolution primarily contributed by highly to partially recessive deleterious mutations. Theories suggest that transient genetic association with fitness alleles can be important in affecting the evolution of alleles that modify the selfing rate during its sweep. Nevertheless, empirical tests often focus on the pre-existing genetic association between selfing rate and ID maintained under mutation–selection balance. Therefore, how this standing genetic association is affected by key factors and its impacts on the evolution of selfing remain unclear. I show that as the selection coefficient of deleterious mutations increases, the association between selfing rate and ID declines from positive to negative. These results predict that association between selfing and ID tends to be negative in populations with low selfing rates, while positive in highly selfing populations. Using population genetic and quantitative genetic models, I show that standing genetic associations between selfing rate and fitness alleles can significantly impact the evolution of the mean selfing rate of a population. I present better metrics of population-level ID, which can be calculated based on the correlation coefficient between individual selfing rate and the fitness of selfed and outcrossed offspring.

## 1 Introduction

Inbreeding depression (ID) is the reduced survival and fertility of offspring produced from inbreeding compared to offspring from mating between unrelated individuals. ID is a major genetic factor that affects the evolution of mating systems, including self-fertilization (Goodwillie et al., 2005) and biparental inbreeding (Uyenoyama, 1986). As inbreeding increases genome-wide homozygosity, there are two primary hypotheses about the genetic basis of ID: increased homozygosity at fitness loci with heterozygotes advantages (overdominance) and increased homozygosity of partially recessive deleterious mutations. While these hypotheses are not mutually exclusive, empirical evidence suggests that ID may be mainly contributed by partially recessive deleterious mutations (Charlesworth and Charlesworth, 1999; Charlesworth and Willis, 2009). Furthermore, empirical estimations suggest that deleterious mutations have a continuous distribution in fitness effects ranging from strongly to slightly deleterious, and mutations with larger-effect tend to be more recessive (Eyre-Walker and Keightley, 2007; Charlesworth and Willis, 2009; Charlesworth, 2012). Although the genomic mutation rate of small-effect deleterious mutations tends to be higher than large-effect ones (Mukai et al., 1972; Simmons and Crow, 1977; Klekowski and Godfrey, 1989), large-effect mutations may have a substantial contribution to ID due to high recessiveness (Lande et al., 1994; Porcher and Lande, 2005; Winn et al., 2011).

Selfing is a major form of inbreeding in plants (Jarne and Charlesworth, 1993), and the impacts of ID on the evolution of selfing have received long-standing attention. Compared to outcrossers, selfing individuals can contribute 50% more gametes to the next generation by providing pollen for both other individuals’ and their own ovules. This transmission advantage is weaker if selfing reduces the amount of exported pollen (pollen discounting; Harder and Wilson, 1998). By treating ID as a fixed parameter, early models show that without pollen discounting, selfing is favored when the fitness of selfed offspring relative to outcrossed offspring exceeds 0.5 (Lloyd, 1979; Lande and Schemske, 1985).

Subsequent models relaxed the assumption of fixed ID by considering the joint evolution of selfing and deleterious mutations. These studies found that genetic associations between alleles modifying the selfing rate and alleles affecting fitness can be important in mediating the evolution of selfing (Holsinger, 1988; Uyenoyama and Waller, 1991a, 1991b, 1991c). A modifier enhancing selfing can develop association with fitter alleles by promoting segregation and, thus, may invade even when ID is high (Holsinger, 1988; Uyenoyama and Waller, 1991a, 1991c). Conversely, in a highly selfing population, an outcrossing-enhancing modifier may invade under low ID (Kamran-Disfani and Agrawal, 2014; Xu, 2022) because outcrossing promotes effective recombination between loci, thus increasing the efficacy of selection (Uyenoyama and Waller, 1991a). However, that genetic associations with fitness alleles may only slightly affect the invasibility of a selfing rate modifier, unless the modifier or deleterious mutations have strong effects (Charlesworth et al., 1991, 1992; Schultz and Willis, 1995; Damgaard, 1996).

Given the potential importance of genetic associations between selfing rate and fitness alleles during mating system evolution, several studies have sought to identify this correlation in nature, as summarized in **Table 1**. In general, the results are mixed. Some studies found a negative correlation between ID and selfing rate indicating that selfing-enhancing modifiers tend to be associated with fitter alleles, while other studies found positive or no relationship.

**Table 1:** Summary of results of previous studies on the association between inbreeding depression (ID) and selfing rate.

Reference	Species	Mean selfing rate	Trait related to selfing rate	Correlation between ID and selfing rate
Carr et al. (1997)	*Mimulus guttatus*	Low	Herkogamy	Positive, but statistically insignificant
Mutikainen and Delph (1998)	*Lobelia siphilitica*	Low (gynodioecy)	Female vs. hermaphrodite	Positive, but statistically insignificant
Chang and Rausher (1999)	*Ipomoea purpurea*	Low	Herkogamy	Negative
Vogler et al. (1999)	*Campanul rapunculoides*	Low	Strength of self-incompatibility	Negative
Takebayashi and Delph (2000)	*Gilia achilleifolia*	Low	Herkogamy	Negative
Rao et al. (2002)	*Brassica cretica*	Low	Strength of self-incompatibility	No correlation
Stone and Motten (2002)	*Datura stramonium*	High	Herkogamy	Negative
Sebastián Escobar et al. (2007)	*Physa acuta* ^a^	Low	Waiting time	No correlation
Jiménez-Lobato and Núñez-Farfán (2021)	*Datura inoxia*	Intermediate	Herkogamy	Positive^b^

a Aquatic gastropod.

b Inbreeding depression is estimated based on the difference between the inbreeding coefficient F within the adult cohort and F within the progeny cohort.

Nevertheless, theoretical models and the above empirical tests actually focus on different types of genetic associations and, thus, are not directly comparable. Theoretical studies look at the transient genetic association developed during the sweep of selfing rate modifier (Charlesworth et al., 1991; Uyenoyama and Waller, 1991a). This association is observable only in a short period since the modifiers sweep rapidly (Schultz and Willis, 1995). In contrast, what the empirical studies detected is the standing genetic association formed between fitness alleles and segregating selfing rate modifiers under mutation–selection balance. Therefore, to better understand the empirical results, we need theoretical analyses of standing genetic associations between selfing rate and ID in a population with selfing rate variation maintained under selection–mutation balance.

More importantly, the association between selfing rate and ID raises the question on how population-level ID should be defined and measured. Empirical studies usually estimate the population-level ID either by averaging the fitness of selfed and outcrossed offspring, or by averaging the family-level ID, referred to as population and family ID, respectively, by Johnston and Schoen (1994). Nevertheless, it is questionable whether this average-level metric truly reflects the strength of selection on selfing caused by deleterious mutations, especially when there is association between selfing rate and ID.

Here, I first show that standing genetic association between selfing rate and ID at equilibrium depends on the selection coefficient of deleterious mutations, which decreases from positive to negative as the selection coefficient becomes larger. I then show how genetic association between selfing rate and fitness alleles can impact the evolution of selfing under two scenarios: the invasion of a rare selfing rate modifier, and the evolution of the mean selfing rate from standing variation when treating selfing rate as a quantitative trait. I discuss how the population-level ID can be better measured in future empirical studies.

## 2 Methods

I use individual-based simulations to investigate the genetic association between the selfing rate and deleterious mutations in a population at selection–mutation–drift balance (C++ code is available at https://doi.org/10.5281/zenodo.10702627). Briefly, the simulation considers a hermaphroditic diploid population with non-overlapping generations and a constant population size *N*. Selfing rate is determined by a quantitative trait (e.g., herkogamy) controlled by multiple loci. Deleterious mutations occur at an infinite number of loci, which are linked with selfing-related loci. The level of linkage is determined by the total number of crossovers in the genome. Each generation starts with the adult population, followed by reproduction, mutation, and viability selection of the offspring.

Each individual carries two chromosomes with the length scaled to be 1. I assume the individual selfing rate *α* depends on the phenotype of a quantitative trait *z* as $\alpha =\frac{1}{1+e^{-k\left(z-z_{c}\right)}}$ (Degottex-Féry and Cheptou, 2023). The parameter *k* determines sensitivity of the selfing rate to the phenotype *z*. The parameter *z_c_* is the phenotype at which the selfing rate is *α* = 0.5, which is altered to change the mean selfing rate of the population. I assume phenotype *z* is controlled by *n_z_* identical loci, with their positions on the chromosome being 0,1/*n_z_*,…, $\left(n_{z}-1\right)/n_{z}$ . The positions of selfing-related loci should not qualitatively affect the results, as will be discussed in the Discussion section. Each locus has two alleles *A* and *a* with additive effects. Allele *A* and *a* change the phenotypic value by – $1/\sqrt{n_{z}}$ and $1/\sqrt{n_{z}}$ , respectively. Here the effect of each locus is scaled by $1/\sqrt{n_{z}}$ to eliminate the dependency of genetic variance on the number of loci *n_z_*. The mean genomic mutation rate of the trait is *U_z_* per generation.

Since the trait is subject to selfing-related selective forces exerting directional selection (e.g., transmission advantage and ID; Lande and Schemske, 1985) to maintain genetic variation, I assume the trait is also under stabilizing viability selection. Specifically, the viability of individuals with phenotype *z* is $w_{1}\left(z\right)=e^{-\lambda z^{2}}$ , where *λ* determines the strength of selection. Large values of *λ* are chosen to make viability selection strong enough to outweigh selective forces related to selfing. This ensures that the phenotypic distribution of trait *z* remains nearly the same when we investigate the effects of different parameter values of key factors, such as selection coefficient of deleterious mutations.

I assume deleterious mutations occur at an infinite number of loci (Kondrashov, 1985) and have identical selection coefficient *s* and dominance *h*. Each generation, the number of new mutations on each chromosome, is drawn from a Poisson distribution with parameter *U*/2, with their positions being drawn from a uniform distribution ~U(0,1) by excluding the positions of loci controlling the trait. The fitness component contributed by deleterious mutations is $w_{2}{\left(1-hs\right)}^{n_{het}}{\left(1-s\right)}^{n_{hom}}$ , where *n_het_* and *n_hom_* are the number of deleterious mutations in heterozygous and homozygous states. The overall individual fitness is thus $w=w_{1}w_{2}$ .

To generate the adult population after viability selection, I sample *N* individuals from the juvenile population with replacement, with the probability being proportional to individual fitness. To generate the offspring population, for each offspring, I first randomly sample a parent individual *i* from the adult population and obtain its selfing rate *α_i_*. To determine whether the offspring is produced from self-fertilization or outcrossing, a random number *ϵ* is generated from uniform distribution U(0,1). When *ϵ* > *α_i_*, the offspring is produced by selfing. When *ϵ*< *α_i_*, the offspring is produced by outcrossing, and a second parent *j* ≠ *i* is sampled. To obtain gametes generated by meiosis, the number of crossovers between two chromosomes is drawn from a Poisson distribution with parameter *L*, and the position of each crossover is drawn from U(0,1).

During the simulation, family-level ID is estimated by generating an outcrossed and a selfed offspring from each adult individual, and calculating the fitness of the selfed relative to the outcrossed offspring contributed by deleterious mutations (the calculation is described two paragraphs prior). The population-level ID is calculated as the reduction of the average fitness of selfed offspring relative to the average fitness of outcrossed offspring. For each parameter, I run the population for 5,000 generations, which is sufficiently long for the population to reach the selection–mutation–drift balance. I then calculate the average of each tracked metric over the last 2,000 generations.

## 3 Results

### 3.1 Associations between selfing rate and fitness alleles

**Figure 1:**
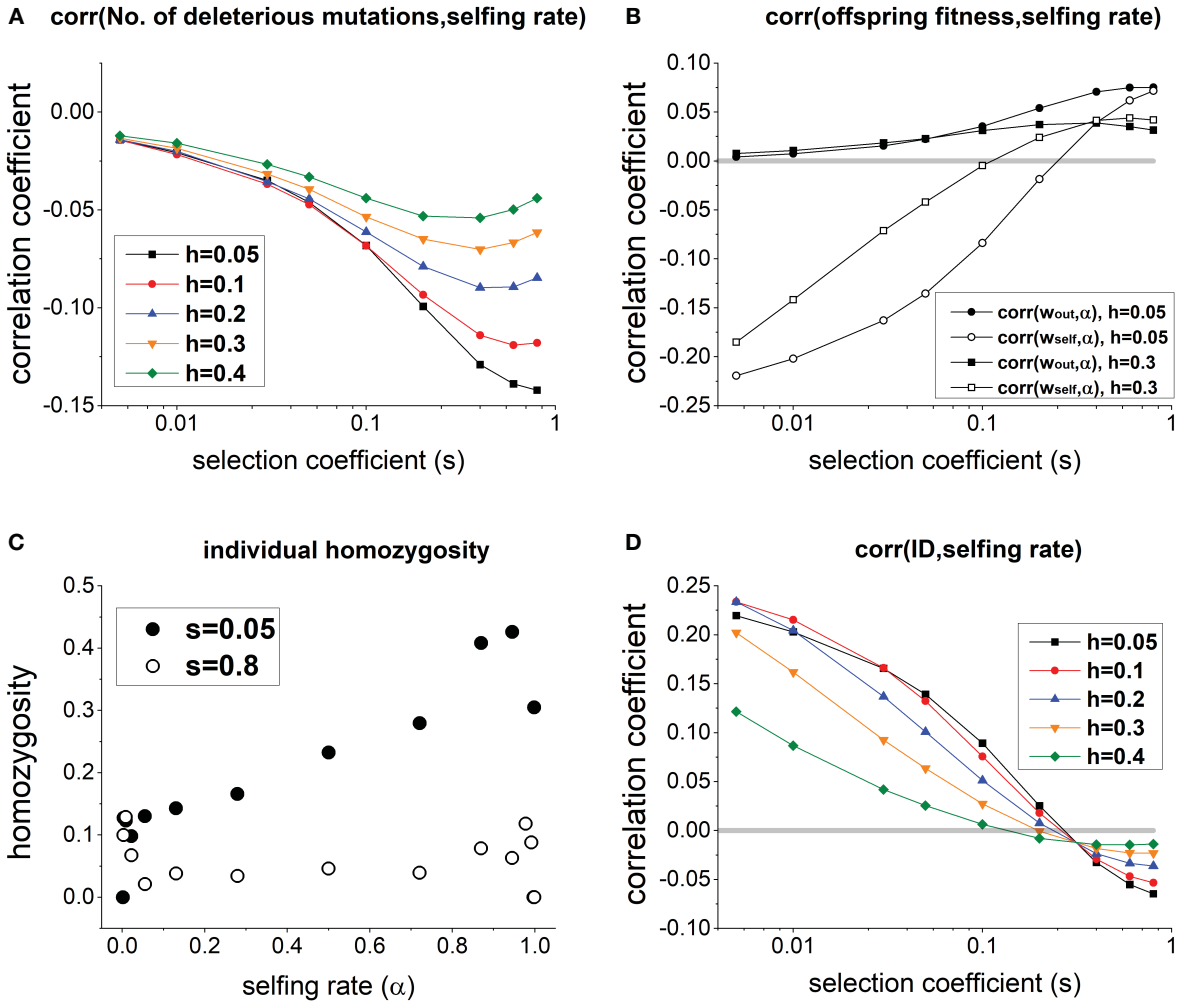
**(A)** Effects of selection coefficient *s* on the correlation between selfing rate and the number of deleterious mutations per individual in a population. **(B)** Effects of *s* on the correlation between selfing rate and the fitness of outcrossed and selfed offspring. **(C)** Changes of the average individual homozygosity with individual selfing rate in a population (note that there are only few individuals when *α* is close to 0 or 1). **(D)** Effects of *s* on the correlation between selfing rate and family-level inbreeding depression measured as $1-w_{self}/w_{out}$ . In **(C)**, *h* = 0.1. Other parameters used are $N=20000,U=0.5,L=10,n_{s}=10,U_{z}=0.2,k=3,\lambda =0.5,z_{c}=0$ .

In a population at equilibrium, individuals with a higher selfing rate generally carry fewer deleterious mutations (**Figure 1A**), but it does not necessarily imply a lower ID. Specifically, due to fewer deleterious mutations, fitness of outcrossed offspring *w_out_* is higher for individuals with a higher selfing rate *α* [corr(*w_out_*,*α*) > 0; solid circles and squares in **Figure 1B**]. However, a higher selfing rate is associated with a lower fitness of selfed offspring *w_self_* [corr(*w_self_*,*α*) < 0] when deleterious mutations have small selection coefficient *s*, and the association becomes positive only when *s* is large enough (see open circles and squares in **Figure 1B**). This is because when *s* is small, individuals with a higher selfing rate *α* exhibit significantly greater homozygosity compared to those with lower *α* (see *s* = 0.05 in **Figure 1C**). Consequently, although individuals with higher *α* carry fewer deleterious mutations, their selfed offspring tend to be less fit due to high homozygosity. In contrast, when *s* is large, homozygosity remains low and only slightly increases with individual selfing rate (see *s* = 0.8 in **Figure 1C**), since deleterious mutations in the homozygous state is strongly selected against.

Therefore, as *s* increases, the correlation coefficient between ID and selfing rate declines from positive to negative (**Figure 1D**). There exists a critical selection coefficient at which the correlation between selfing rate and ID is 0, which is greater when deleterious mutations are more recessive (compare lines with different values of *h* in **Figure 1D**).

The above results are qualitatively robust under different values of the genomic mutation rate of deleterious mutations (**Figure 1A**), population size (**Supplementary Figures 2, 3**), the number of crossovers (**Supplementary Figures 1B, 3**), and the mean selfing rate (**Supplementary Figure 4**). In general, varying parameter values of these factors only slightly alter the magnitude of genetic correlations.

### 3.2 Influences of selfing rate variation and genetic associations on the evolution of selfing

I consider two scenarios to illustrate how selfing rate variation and genetic associations between selfing rate modifiers and deleterious mutations may impact the evolution of selfing. For simplicity, I assume selfing does not reduce pollen exported to fertilize other individuals (i.e., no pollen discounting). In the first scenario, I consider a selfing rate modifier locus with two alleles *M* and *m*, and I focus on the condition for a rare modifier allele *m* to invade in a population previously fixed with allele *M*. In the second scenario, I treat selfing rate as a quantitative trait and investigate the evolution of the mean selfing rate in a population from standing variation.

#### 3.2.1 Invasion of a selfing rate modifier

I denote the fitness of selfed and outcrossed offspring of an individual with selfing rate *α* by *w_self_* (*α*) and *w_out_* (*α*), respectively. The mean fitness of allele *M* is thus


(1) $$\begin{array}{c} w_{M}=E[\alpha w_{self}\left(\alpha \right)+\frac{1}{2}\left(1-\alpha \right)w_{out}\left(\alpha \right)+\frac{1}{2}E\left[\left(1-\alpha \right)w_{out}\left(\alpha \right)\right]], \end{array}$$

In Equation (1), the first term represents the contribution from selfed offspring. The second term accounts for the fitness contributed by outcrossed ovules. The third term captures the contribution from exported pollen that sires other individuals. For an *MM* individual with selfing rate *α*, I assume the replacement of allele *M* by allele *m* (genotype *Mm*) change the selfing rate by *f*(*α*). How *f*(*α*) may change with the selfing rate *α* should depend on the specific mechanism that the modifier alters the selfing rate. For example, if the modifier reduces the strength of self-incompatibility, individuals receiving a higher proportion of self pollen may have a larger selfing rate increase than those previously receiving more non-self pollen. Thus, *f*(*α*) increases with *α*. In contrast, if the modifier changes selfing-related phenotypes (e.g., flower size), individuals with a higher selfing rate may have a smaller increase in the selfing rate due to diminishing return, so that *f*(*α*) is a decreasing function of *α*.

Assuming that the rare modifier allele *m* occurs randomly in different genetic backgrounds, its expected fitness is


(2) $$\begin{array}{c} w_{m}=E\left[\left(\alpha +f\left(\alpha \right)\right)w_{self}\left(\alpha \right)+\frac{1}{2}\left(1-\left(\alpha +f\left(\alpha \right)\right)\right)w_{out}\left(\alpha \right)+\frac{1}{2}E\left[\left(1-\alpha \right)w_{out}\left(\alpha \right)\right]\right]\\ \begin{array}{c} =w_{M}+\left(\frac{E\left[w_{self}\left(\alpha \right)f\left(\alpha \right)\right]}{E\left[w_{out}\left(\alpha \right)f\left(\alpha \right)\right]}-\frac{1}{2}\right)E[w_{out}\left(\alpha \right)f\left(\alpha \right)], \end{array} \end{array}$$

Equation (2) suggest that ID can be defined as $\delta ≜1-\frac{E\left[w_{self}\left(\alpha \right)f\left(\alpha \right)\right]}{E\left[w_{out}\left(\alpha \right)f\left(\alpha \right)\right]}$ . Therefore, when the modifier *m*, on average, increases the selfing rate ( $E\left[w_{out}\left(\alpha \right)f\left(\alpha \right)\right]>0$ ), it invades when *δ* < 0.5.

Some approximations of Equation (2) are useful to illustrate how selfing rate variation and genetic associations affect the invasion condition. Assuming that the fitness of selfed and outcrossed offspring changes linearly with their mother’s selfing rate *α* (as supported by **Supplementary Figure 5**), the **Supplementary Materials** show that population-level ID is


(3) $$\begin{array}{c} \delta \approx 1-\frac{E\left[w_{self}\left(\alpha \right)\right]+\rho_{self}V_{\alpha }f^{\prime }\left(\bar{\alpha }\right)/f\left(\bar{\alpha }\right)}{E\left[w_{out}\left(\alpha \right)\right]+\rho_{out}V_{\alpha }f^{\prime }\left(\bar{\alpha }\right)/f(\bar{\alpha }),} \end{array}$$

In Equation (3), $\bar{\alpha }$ is the mean selfing rate, and *ρ_self_* is the correlation coefficient between parental selfing rate and the fitness of selfed offspring (similar for *ρ_out_*). When *f*(*α*) is constant, Equation (3) becomes $1-\frac{E\left[w_{self}\left(\alpha \right)\right]}{E\left[w_{out}\left(\alpha \right)\right]}$ , and I denote ID in this baseline case by *δ*_0_, which is exactly the population-level ID calculated by empirical studies using. In this case, genetic associations between selfing rate modifiers and fitness alleles have no effect on the evolution of a selfing rate modifier. Therefore, provided that the selfing rate modifier changes the selfing rate of all individuals by the same amount or there is no genetic association (*ρ_self_* = *ρ_out_* = 0), *δ*_0_ is unbiased in reflecting the selective strength on a selfing rate modifier caused by deleterious mutations.

**Figure 2:**
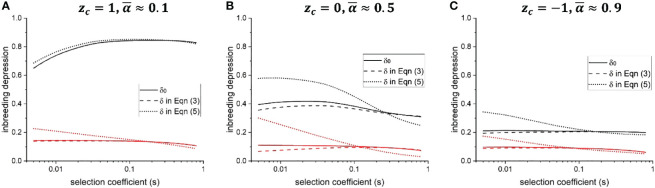
Effects of genetic associations between selfing rate modifiers and fitness alleles on the population-level inbreeding depression. Solid line: baseline ID, defined as $\delta_{0}=1-E\left[w_{self}\left(\alpha \right)\right]/E\left[w_{out}\left(\alpha \right)\right]$ ; dashed line: ID defined in Equation (3), assuming *f*(*α*) is proportional to the outcrossing rate 1–*α*, thus $f^{\prime }\left(\bar{\alpha }\right)/f\left(\bar{\alpha }\right)=-\frac{1}{1-\alpha }$ ; dotted line: ID defined in Equation (5). Black and red color show results for *h* = 0.05 and *h* = 0.3, respectively. **(A–C)** show results when the mean selfing rate is low, intermediate, and high, respectively. Parameters used for calculating ID are obtained from individual-based simulations. Other parameters used in the individual-based simulations are $N=20000,U=0.5,L=10,n_{s}=10,U_{z}=0.2,k=3,\lambda =0.5$ .

When *f*(*α*) changes with *α*, suppose that the selfing rate increase is smaller for individuals with a higher background selfing rate (i.e., $f^{\prime }\left(\bar{\alpha }\right)<0$ ). By Equation (3), *δ* < *δ*_0_ when $\frac{\rho_{self}}{\rho_{out}}<\frac{E\left[w_{self}\left(\alpha \right)\right]}{E\left[w_{out}\left(\alpha \right)\right]}$ , so that genetic associations between selfing rate and fitness alleles promote the invasion of a selfing-enhancing modifier. In contrast, the genetic associations inhibit the evolution of selfing (*δ* > *δ*_0_) when $\frac{\rho_{self}}{\rho_{out}}>\frac{E\left[w_{self}\left(\alpha \right)\right]}{E\left[w_{out}\left(\alpha \right)\right]}$ . **Figure 1A** suggests that *ρ_self_*/*ρ_out_* tends to increase as selection coefficient of deleterious mutations *s* becomes larger. Therefore, genetic associations will promote the invasion of a selfing-enhancing modifier (*δ*< *δ*_0_) when *s* is small, while inhibiting its invasion (*δ* > *δ*_0_) when *s* is large (compare solid and dashed lines in **Figure 2**).

In general, the difference between ID defined by Equation (3) and the baseline ID *δ*_0_ is often slight (**Figure 2**; **Supplementary Figure 6**), unless the population size is small, and deleterious mutations have weak effects (**Supplementary Figure 7**). This is because the correlation between offspring fitness and selfing rate, *ρ_self_* and *ρ_out_*, are often smaller than 0.1 (**Figure 1A**), and the variance of selfing rate in a population *V_α_* cannot exceed 0.25. Therefore, in Equation (3), the difference between *δ* and *δ*_0_ caused by genetic associations is slight, unless selfing rate modification is highly sensitive to the individual selfing rate (i.e., the term $f^{\prime }\left(\bar{\alpha }\right)/f\left(\bar{\alpha }\right)$ is large).

#### 3.2.2 Evolution of the mean selfing rate

When the selfing rate is a quantitative trait, the evolution of the mean selfing rate is determined by the selection gradient (Lande, 1979). Based on Equation (1), the selection gradient is


(4) $$\begin{array}{c} E\left[\frac{\partial w\left(\alpha \right)}{\partial \alpha }\right]=E\left[w_{self}\left(\alpha \right)-\frac{1}{2}w_{out}\left(\alpha \right)+\alpha w_{self}^{'}\left(\alpha \right)+\frac{1}{2}\left(1-\alpha \right)w_{out}^{'}\left(\alpha \right)\right]\\ \begin{array}{c} \approx w_{out}\left(\bar{\alpha }\right)\left(\frac{w_{self}\left(\bar{\alpha }\right)+\bar{\alpha }\rho_{self}+\frac{1}{2}\left(1-\bar{\alpha }\right)\rho_{out}}{w_{out}\left(\bar{\alpha }\right)}-\frac{1}{2}\right) \end{array} \end{array}$$

where the last expression uses the linear approximation for *w_self_* (*α*) and *w_out_* (*α*). Equation (4) suggests that population-level ID can be defined as


(5) $$\begin{array}{c} \delta ≜1-\frac{w_{self}\left(\bar{\alpha }\right)+\bar{\alpha }\rho_{self\;}+\frac{1}{2}\left(1-\bar{\alpha }\right)\rho_{out}}{w_{out}\left(\bar{\alpha }\right)} \end{array}$$

so that the mean selfing rate evolves to be higher when *δ* < 0.5.

*δ* defined in Equation (5) can significantly deviate from the baseline ID *δ*_0_ unless the population is predominantly outcrossing (compare solid and dotted lines in **Figure 2**), or the rate of recombination across the genome is low (**Supplementary Figure 6B**). Whether *δ* is higher or lower than *δ*_0_ depends on the selection coefficient of deleterious mutations *s*. When *s* is small, *δ* is much larger than the baseline ID *δ*_0_, so that genetic associations between selfing rate modifiers and fitness alleles prevent the mean selfing rate from evolving to be higher. When *s* is large, *δ* < *δ*_0_, so genetic associations promote the evolution of a higher mean selfing rate.

## 4 Discussion

This study investigates the following two questions: (1) how key genetic factors affect the standing genetic associations between selfing rate and deleterious mutations at equilibrium and (2) how to measure population-level inbreeding depression to incorporate the impacts of this genetic association on the evolution of selfing.

Although individuals with a higher selfing rate will carry fewer deleterious mutations, it does not mean they will have lower ID, and it is more informative to measure the association between selfing rate and the fitness of selfed and outcrossed offspring. In fact, the association between selfing rate and family-level ID will decrease from positive to negative as the selection coefficient of deleterious mutations *s* increases. This is because when *s* is small, due to higher genome-wide homozygosity, individuals with a higher selfing rate will have less fit selfed offspring but more fit outcrossed offspring. When *s* is large, both selfed and outcrossed offspring of individuals with higher selfing rate are fitter.

The model thus predicts that the correlation at equilibrium between selfing rate and family-level ID tends to be negative in populations with a low mean selfing rate, but positive in highly selfing population. Results from previous studies are equivocal in supporting this prediction (see **Table 1**) perhaps because the basis of ID differs across species. Specifically, highly recessive, large-effect mutations can be effectively purged when the selfing rate is high enough, but partially recessive, weak-effect mutations are hard to be purged (Lande and Schemske, 1985; Lande et al., 1994). Therefore, large-effect mutations, which cause a negative association between selfing rate and ID, should contribute more to ID in predominantly outcrossing populations than highly selfing populations (Winn et al., 2011). The correlation may be weakest in populations with intermediate selfing rates, since small- and large-effect mutations may have comparable contributions to ID.

More importantly, the current model reveals that standing genetic associations between selfing rate modifiers and fitness alleles will impact the evolution of selfing. The average-level inbreeding depression, commonly computed in empirical studies (Johnston and Schoen, 1994), is valid in predicting whether a selfing rate modifier can invade or not, but it can be often greatly biased in determining the evolution of the mean selfing rate.

To better measure population-level inbreeding depression, it is useful to first estimate the correlation coefficient between individual selfing rate (or a phenotype related to the selfing rate) and the fitness of selfed and outcrossed offspring. Two metrics of population-level inbreeding depression can then be calculated based on Equations (3) and (5), and the values can be compared with the average-level inbreeding depression. Nevertheless, individual-based simulations suggest that there can be large variation in family-level inbreeding depression among individuals with the same selfing rate, due to variation in the number of new mutations and inbreeding history (Kelly, 2005). Large variation in family-level inbreeding depression can render the genetic correlations statistically insignificant, as found in several previous studies (see **Table 1**).

In addition, although the current simulation assumes that selfing-related loci are distributed evenly along the chromosome, the results should be qualitatively robust to the position of these loci. For intuition, note that the results are independent of the positions when all loci are nearly free recombining or completely linked. The positions of loci may make a difference only when the number of crossovers along the chromosome is intermediate, and the results should be at the intermediate between the results when the number of crossovers along the chromosome is low and high. Nevertheless, it is found that results from individual-based simulations are qualitatively similar when the number of crossovers is low and high (**Supplementary Figures 1, 3**). Therefore, the position of loci should not qualitatively affect the conclusions above.

## Data availability statement

The original contributions presented in the study are included in the article/**Supplementary Material**. Further inquiries can be directed to the corresponding author.

## Author contributions

KX: Conceptualization, Formal analysis, Funding acquisition, Investigation, Methodology, Project administration, Resources, Software, Validation, Visualization, Writing – original draft, Writing – review & editing.
