# Supplementary material for: How should we measure population-level inbreeding depression? Impacts of standing genetic associations between selfing rate and deleterious mutations
Source: Front Plant Sci. 2024 Jul 9;15:1379730. doi: 10.3389/fpls.2024.1379730 (PMC11263115; doi:10.3389/fpls.2024.1379730)
Supplement: Supplementary file 1 [file DataSheet_1.pdf]

Supplementary Materials for "How should we measure population-level inbreeding depression? Impacts of standing genetic associations between selfing rate and deleterious mutations"

1. Supplementary Materials

Here I show the derivation for equation (3) in the main text. Expanding, the selfing rate change by the modifier to the first order as $f(\alpha) \approx f(\overline{\alpha}) + f'(\overline{\alpha})(\alpha - \overline{\alpha})$, the term $\frac{\mathbb{E}[w_{self}(\alpha)f(\alpha)]}{\mathbb{E}[w_{out}(\alpha)f(\alpha)]}$ in equation (2) becomes

$$\frac{\mathbb{E}\left[w_{self}(\alpha)f(\alpha)\right]}{\mathbb{E}[w_{out}(\alpha)f(\alpha)]} \approx \frac{\mathbb{E}\left[w_{self}(\alpha)\left(f(\overline{\alpha}) + f'(\overline{\alpha})(\alpha - \overline{\alpha})\right)\right]}{\mathbb{E}\left[w_{out}(\alpha)\left(f(\overline{\alpha}) + f'(\overline{\alpha})(\alpha - \overline{\alpha})\right)\right]}$$
$$= \frac{f(\overline{\alpha})\mathbb{E}\left[w_{self}(\alpha)\right] + f'(\overline{\alpha})\mathbb{E}\left[w_{self}(\alpha)(\alpha - \overline{\alpha})\right]}{f(\overline{\alpha})\mathbb{E}[w_{out}(\alpha)] + f'(\overline{\alpha})\mathbb{E}[w_{out}(\alpha)(\alpha - \overline{\alpha})]}. \tag{S1}$$

Under the linear approximation for the fitness of salted and outcrossed offspring, $w_{self}(\alpha) \approx w_{self}(\overline{\alpha}) + \rho_{self}(\alpha - \overline{\alpha}) = \mathbb{E}\left[w_{self}(\alpha)\right] + \rho_{self}(\alpha - \overline{\alpha})$, and $w_{out}(\alpha) \approx w_{self}(\overline{\alpha}) + \rho_{out}(\alpha - \overline{\alpha}) = \mathbb{E}\left[w_{self}(\alpha)\right] + \rho_{self}(\alpha - \overline{\alpha})$, equation (S1) becomes

$$\frac{f(\overline{\alpha})\mathbb{E}\left[w_{self}(\alpha)\right] + f'(\overline{\alpha})\mathbb{E}\left[(\alpha - \overline{\alpha})\left(\mathbb{E}\left[w_{self}(\alpha)\right] + \rho_{self}(\alpha - \overline{\alpha})\right)\right]}{f(\overline{\alpha})\mathbb{E}[w_{out}(\alpha)] + f'(\overline{\alpha})\mathbb{E}\left[(\alpha - \overline{\alpha})\left(\mathbb{E}[w_{out}(\alpha)] + \rho_{out}(\alpha - \overline{\alpha})\right)\right]}$$
$$= \frac{f(\overline{\alpha})\mathbb{E}\left[w_{self}(\alpha)\right] + f'(\overline{\alpha})\rho_{self}\mathbb{E}[(\alpha - \overline{\alpha})^2]}{f(\overline{\alpha})\mathbb{E}[w_{out}(\alpha)] + f'(\overline{\alpha})\rho_{out}\mathbb{E}[(\alpha - \overline{\alpha})^2]}$$
$$= \frac{\mathbb{E}\left[w_{self}(\alpha)\right] + \ln\left(f(\overline{\alpha})\right)'\rho_{self}V_\alpha}{\mathbb{E}[w_{out}(\alpha)] + \ln\left(f(\overline{\alpha})\right)'\rho_{out}V_\alpha}. \tag{S2}$$

2. Supplementary Figures

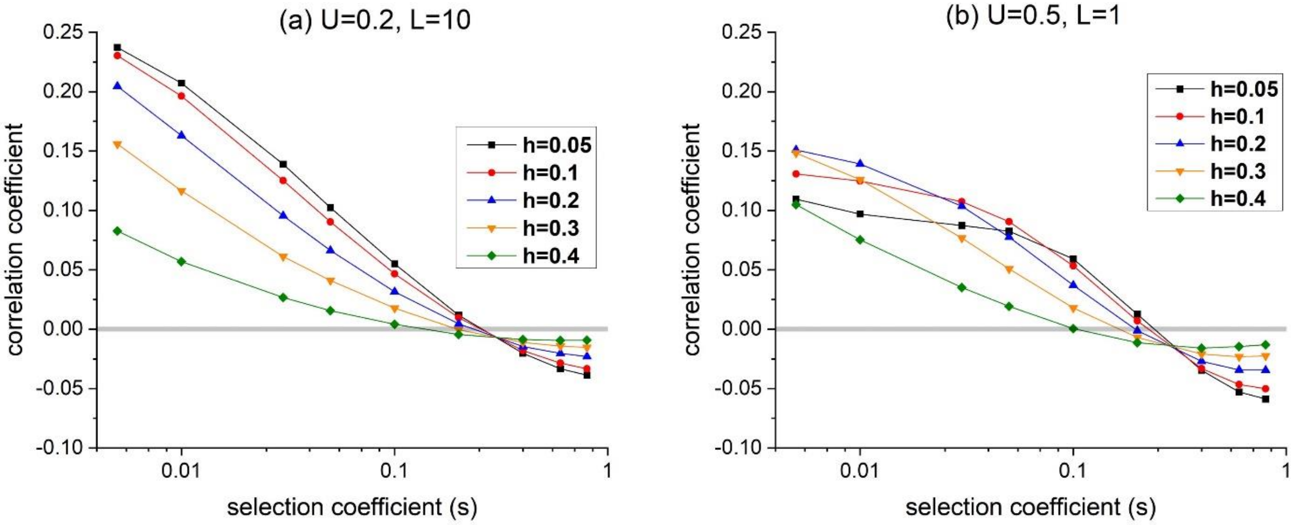

Supplementary Figure 1. Influences of the genomic mutation rate of deleterious mutations U and the number of crossovers on how the correlation between ID and selfing rate. Compared to Fig. 1(d) in the main text with $U = 0.5, L = 10$, the correlation coefficient is lower under a lower genomic mutation rate (panel (a)), under tighter linkage (panel (b)). Other parameters are the same as those used for Fig. 1.

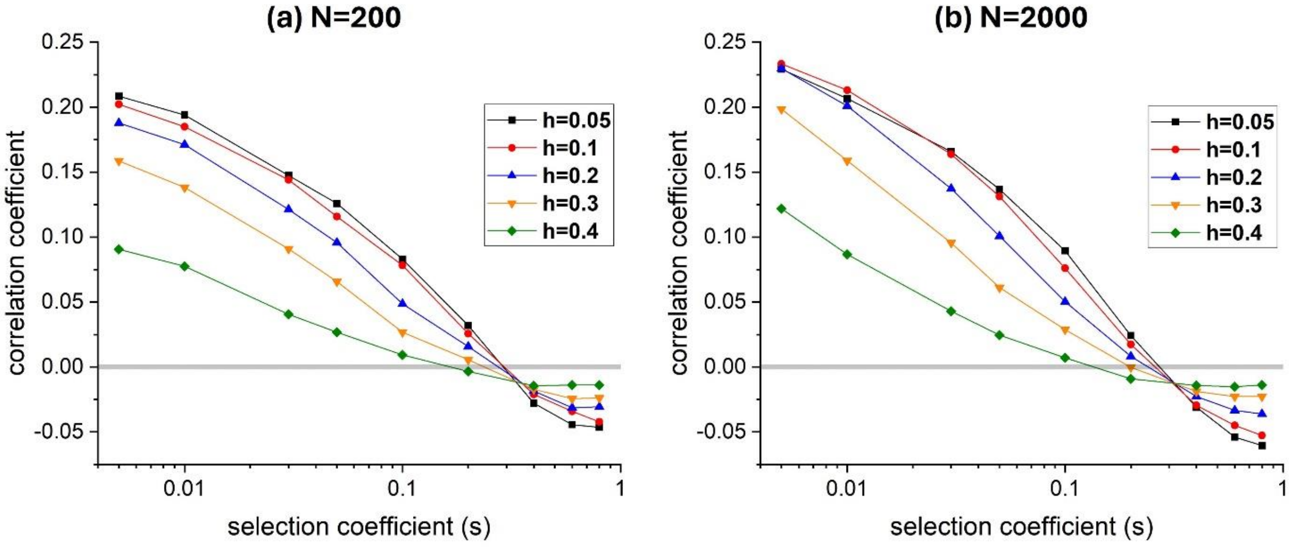

Supplementary Figure 2. Influences of the population size on how the correlation coefficient between ID and selfing rate changes with the selection coefficient of deleterious mutations s. Other parameters are the same as those used for Fig. 1.

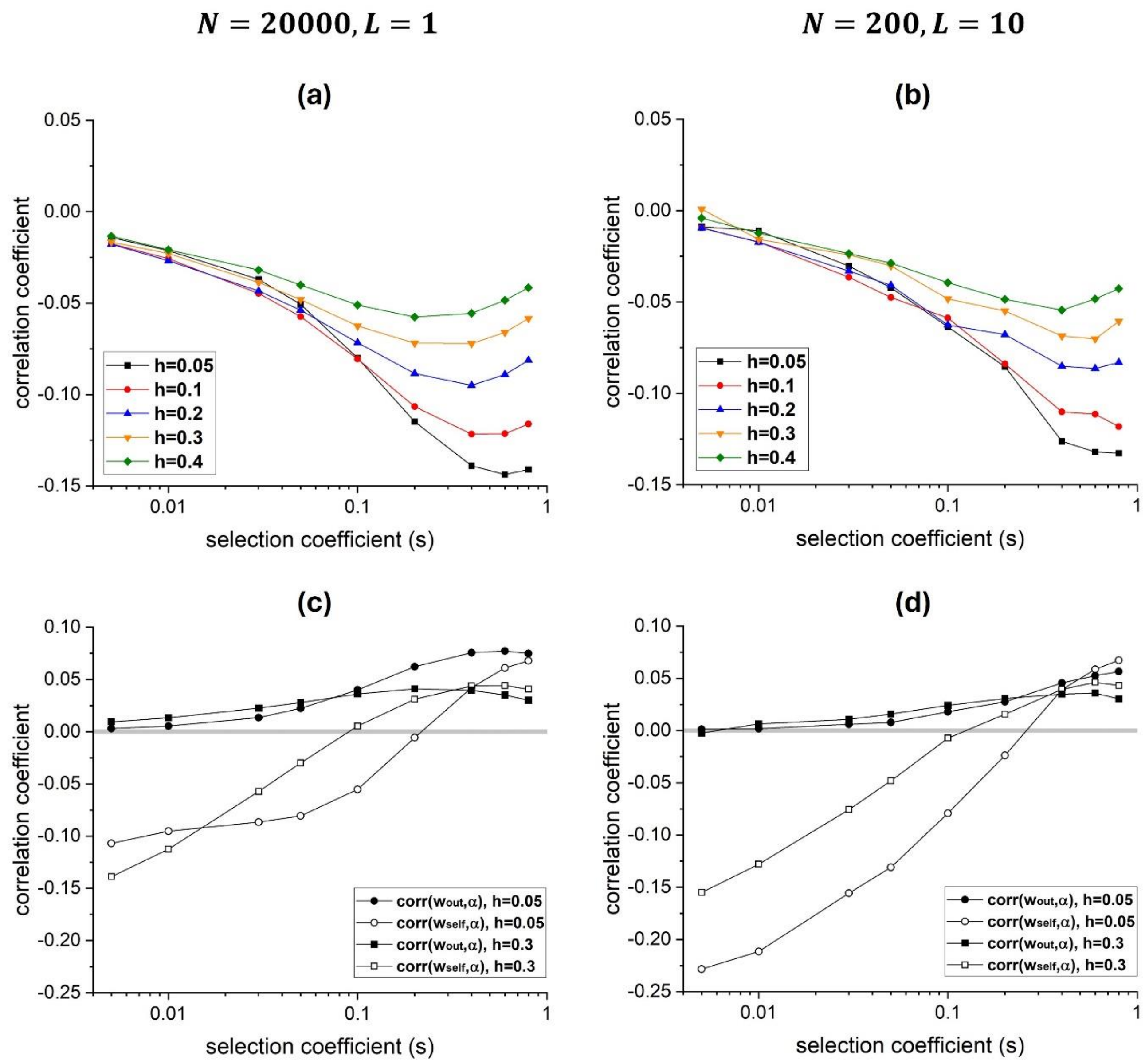

Supplementary Figure 3. Influences of the rate of recombination (left column) and population size (right column) on the correlation between the number of deleterious mutations per individual and selfing (panels (a)-(b)), and the correlation between offspring fitness and selfing rate (panels (c)-(d)). Compared to Figs. 1(a) and 1(b) where $N = 20000, L = 10$, the correlation coefficient is smaller in both panels (a) and (b). Other parameters are the same as those used for Fig. 1.

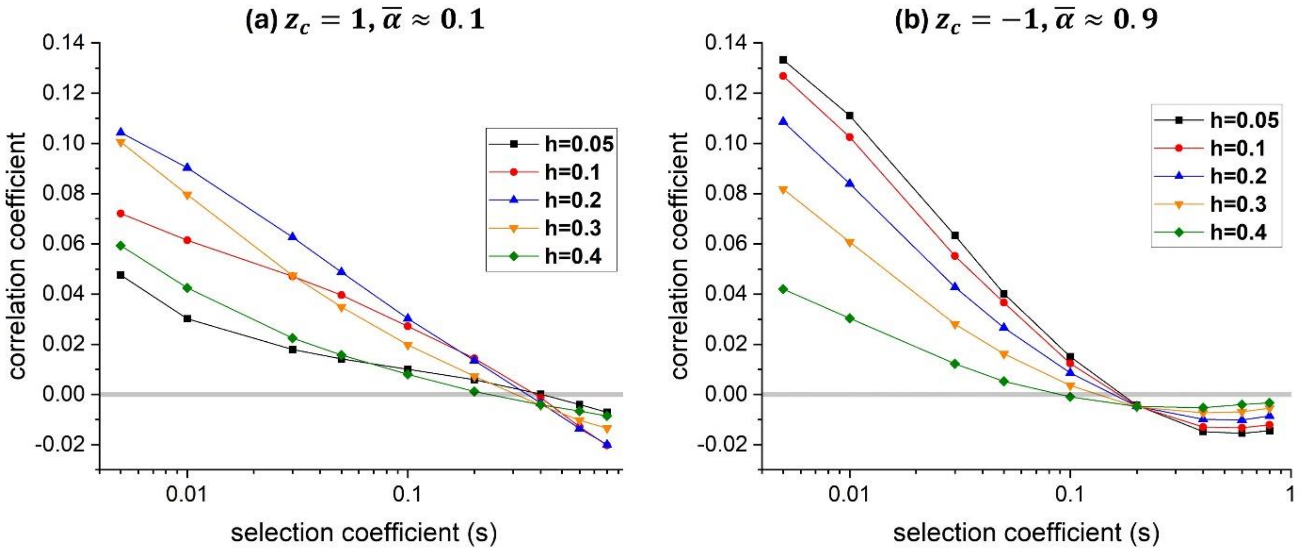

Supplementary Figure 4. Influences of the mean selfing rate of the population $\overline{\alpha}$ on how the correlation coefficient between ID and selfing rate changes with the selection coefficient of deleterious mutations s. The mean selfing rate is changed by changing the parameter z_c described in the method section. Other parameters are the same as those used for Fig. 1.

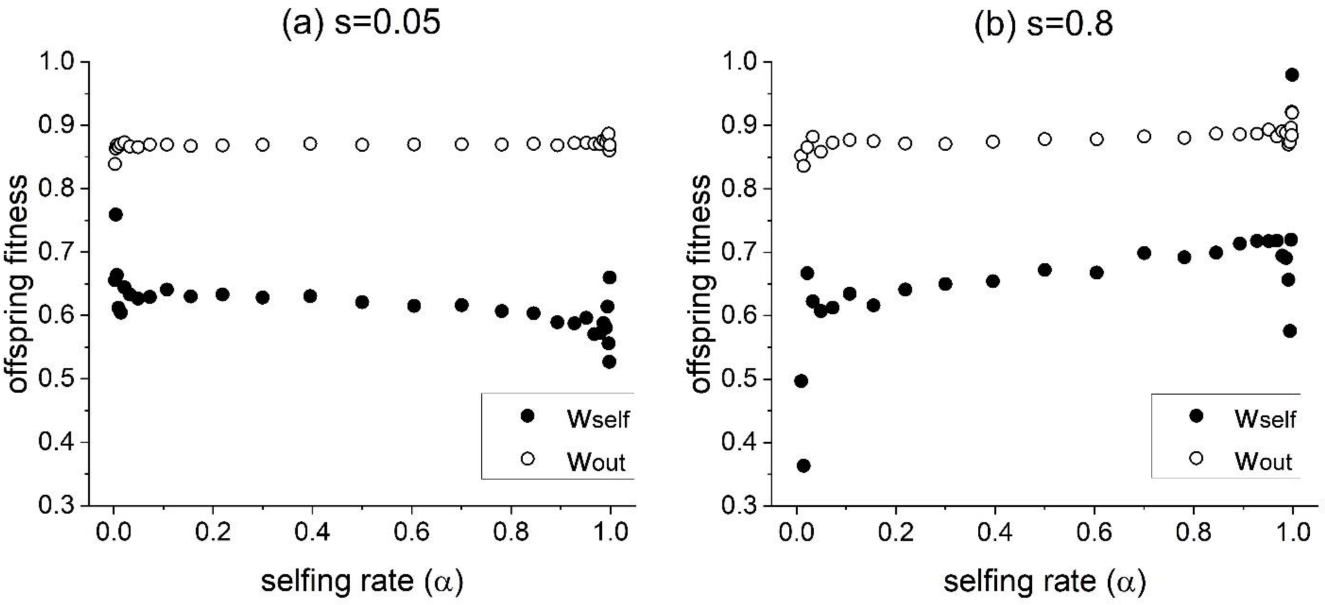

Supplementary Figure 5. Changes of the selfed and outcrossed offspring fitness with the parental selfing rate in a population from individual-based simulations. For each selfing rate α, the offspring fitness is averaged across all individuals with selfing rate being α. Panels (a) and (b) show results when the selection coefficient of deleterious mutations s is small and large, respectively. The dots become scattered when α is close to 0 or 1, since there are only few individuals. Other parameters are $N = 20000, h = 0.1, U = 0.5, L = 10, n_s = 50, U_z = 0.2, k = 3, \lambda = 0.5$, $z_c = 0$.

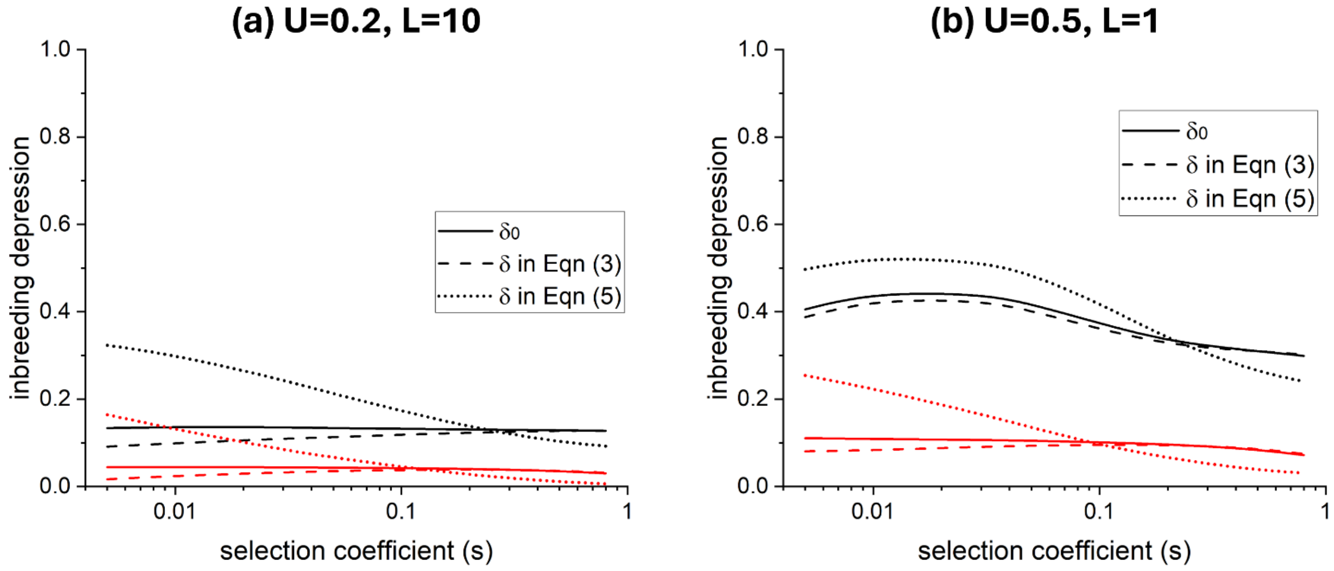

Supplementary Figure 6. Effects of the genomic mutation rate of deleterious mutations (panel (a)) and the number of crossovers (panel (b)) on different metrics of population-level inbreeding depression. Figure legends are described in Fig. 2. Other parameters used are the same as Fig. 2(b).

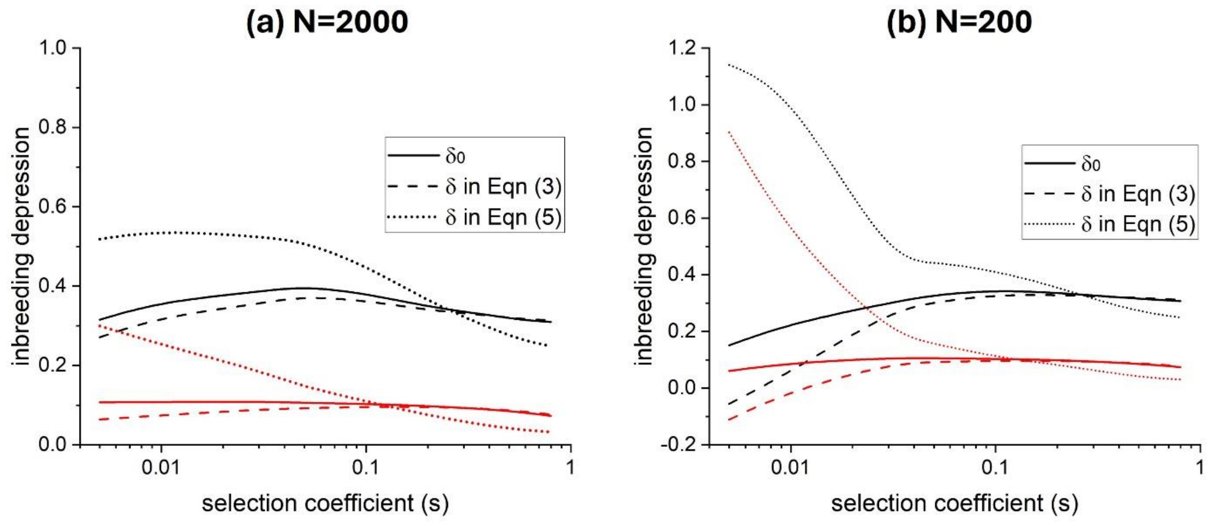

Supplementary Figure 7. Effects of population size on different metrics of population-level inbreeding depression. Figure legends are described in Fig. 2. Other parameters used are the same as Fig. 2(b).
